# The beat goes on

**DOI:** 10.7554/eLife.36882

**Published:** 2018-05-08

**Authors:** Tobias Wang

**Affiliations:** 1 Department of ZoophysiologyAarhus UniversityAarhusDenmark; 2 Aarhus Institute of Advanced StudiesAarhus UniversityAarhusDenmark

**Keywords:** American alligator, conduction system, evolution, heart chambers, atrioventricular node, endothermy, Other

## Abstract

Why is the alligator heart so similar to the hearts of birds and mammals?

**Related research article** Jensen B, Boukens BJ, Crossley DA, Conner J, Mohan RA, van Duijvenboden K, Postma AV, Gloschat CR, Elsey RM, Sedmera D, Efimov IR, Christoffels VM. 2018. Specialized impulse conduction pathway in the alligator heart. *eLife* **7**:e32120. doi: 10.7554/eLife.32120

The human heart consists of four chambers – two atria and two ventricles – that expand and contract in order to drive blood containing oxygen and nutrients throughout the body. The atria, which have relatively thin walls, fill first, before squeezing the blood into the much stronger ventricles, which then contract to send blood coursing through our arteries. Most reptiles have two atria and one ventricle. The only exceptions are the 23 living species of crocodilians (alligators, caimans, crocodiles and gharials) who, like birds and mammals, have four-chambered hearts with two atria and two ventricles (Jones, 1996; Jensen et al., 2014).

In vertebrates, each heartbeat is initiated when a pacemaker region in one of the atria generates an electrical signal. The structure and exact location of the pacemaker region differ amongst species (Jensen et al., 2017), but it is always innervated by the autonomic nervous system. This allows the body to increase or decrease the heart rate in response to metabolic demands (Wang, 2012).

The electrical signal from the pacemaker region spreads rapidly across the cardiac muscle cells of the atria via structures called gap junctions, and this ensures that the entire wall of each atrium contracts almost simultaneously. Neurons called Purkinje fibers are also involved in this process in birds but, in general, the mechanisms responsible for the contraction of the atria are similar in most vertebrates. However, the way in which the electrical signal travels from the atrium to the ventricle differs amongst vertebrates, and the evolution of this pathway has been the focus of considerable attention for many decades (Davies, 1942; Jensen et al., 2012, 2013). Now, in eLife, Vincent Christoffels of the University of Amsterdam and colleagues – including Bjarke Jensen and Bastiaan Boukens as joint first authors – report new and surprising findings about this phenomenon in alligators (Jensen et al., 2018).

Back in the 17th century, William Harvey had already noticed that the atria contract before the ventricles in a number of different animals. This meant that the electrical signal generated in the pacemaker region must somehow be slowed down at the 'border' between the atria and the ventricles. In both mammals and birds, a layer of fibrous fatty tissue – which does not conduct electricity – insulates the ventricles from the atria. The only way that the electrical signal can pass from the atria to the ventricles is via a small structure called the atrioventricular node, which is positioned immediately above the septum that separates the left and right ventricles. When the electrical signal reaches this node, it activates two bundles of neurons (containing His fibers and Purkinje fibers) that swiftly relay the impulse and cause the ventricles to contract simultaneously.

However, in extant reptiles, the common ancestor of both birds and mammals, there does not seem to be an insulating layer or an anatomically defined node (Davies, 1942). Instead, the electrical signal is slowed down by an intricate arrangement of myocardial fibers at the junction between the two atria and the ventricle. In addition, recent studies have been unable to provide any anatomical evidence for a conduction system in the ventricle of reptiles. The electrical signal appears to be conveyed by the internal lining of the heart, which shares molecular signatures with the conduction system found in birds and mammals (Jensen et al., 2012).

While reptiles rely on their environment to maintain their temperature (that is, they are ectothermic), mammals produce their own heat (so they are endothermic). The high levels of metabolism needed to produce enough warmth means that the resting and maximal metabolic rates of mammals and birds are about 10 times higher than those of ectothermic animals (Bennett and Ruben, 1979). The cardiovascular system must keep up with these greater needs by delivering more oxygen to the body. The four-chambered heart provides an efficient solution by keeping oxygenated and non-oxygenated blood separate. The supply of oxygen to the body can also be improved by increasing how often the heart contracts. This requires cardiac structures that quickly conduct electricity, such as the atrioventricular nodes (Burggren et al., 2014).

Jensen et al. – who are based in Amsterdam and labs in the United States and the Czech Republic – combine electrophysiology and gene expression techniques to identify how electric impulses spread across the crocodilian heart, and to characterize the molecular phenotype of the various chambers. The experiments provided unequivocal evidence of an atrioventricular node in crocodilians. Among extant reptiles, crocodilians are the closest living sister group to birds. However, despite their four-chambered hearts and an atrioventricular node, all living crocodilians are clearly ectothermic and have low heart rates like other reptiles (Hillman and Hedrick, 2015; Lillywhite et al., 1999; Joyce et al., 2018).

With their ability to walk with their body off the ground, their peculiar respiratory muscles, their avian-like lungs and various other traits, crocodilians may have once been endothermic (Seymour et al., 2004; Hillenius and Ruben, 2004). According to this hypothesis, they switched to ectothermy when they adopted an entirely aquatic life style and became sit-and-wait predators with intermittent meals separated by long fasting periods. However, if past crocodilians had warm blood and some of the associated heart structures, have extant species lost their His and Purkinje fibers? Would these cells – which support high-speed electrical signals – pose functional problems in animals with very low heart rates?

The fact that crocodilians have an atrioventricular node also sheds light on the evolution of the vertebrate heart. For example, the mere presence of a node and a division between the ventricles may be enough to prevent the electrical signal from 're-entering' the atria (which would disrupt the operation of the heart). These findings may also suggest that a nodal structure allows better fine-tuning of the heart rate by the autonomic nervous system.

The next step is to characterize the electrophysiological properties of the cells in the atrioventricular node of crocodilians. Electrocardiogram recordings would also help to understand the exact timing of cardiac events, while flow and pressure measurements would capture the dynamics of the blood flow. There may still be delightful discoveries awaiting us inside the four chambers of the crocodilian heart.
